# Draft Genome Sequence of *Pseudomonas* sp. Strain MWU12-2323, Isolated from a Wild Cranberry Bog in Truro, Massachusetts

**DOI:** 10.1128/MRA.01387-19

**Published:** 2020-01-09

**Authors:** Ghazal Ebadzadsahrai, Scott Soby

**Affiliations:** a Biomedical Sciences Program, College of Graduate Studies, Midwestern University, Glendale, Arizona, USA; b Biomedical Sciences Program, College of Graduate Studies and College of Veterinary Medicine, Midwestern University, Glendale, Arizona, USA; Georgia Institute of Technology

## Abstract

Exploration of novel environments such as low-pH wild cranberry bog soils yields a rich diversity of bacteria, including *Pseudomonas* spp. Here, we present the draft genome sequence of *Pseudomonas* sp. strain MWU12-2323, isolated from wild cranberry plant rhizosphere. The genome has secondary metabolite genes encoding carbohydrate polymer-degrading enzymes.

## ANNOUNCEMENT

The genus *Pseudomonas* comprises a large number of species that together produce a long list of secondary metabolites that allow it to occupy a diverse range of niches (1), but little is known about newly discovered species and how they influence plant and soil health. This is particularly true for little-studied environments such as the wild cranberry bog soils in the Cape Cod National Seashore, MA, USA. We isolated *Pseudomonas* sp. strain MWU12-2323 from the rhizosphere of wild cranberry plants as part of a culture-dependent survey of bog soil bacteria, and this article reports its genomic sequence.

Soil samples containing roots from wild cranberry plants were plated on King’s medium B (KMB) supplemented with 50 μg ml^−1^ each ampicillin and cycloheximide and grown at 26°C, and a single colony was purified 3 times on KMB agar. Genomic DNA (gDNA) was extracted from overnight KMB broth cultures using a DNeasy blood and tissue kit (Qiagen). gDNA was sheared to ∼600 bp, and libraries were generated for Illumina sequencing (Apollo 384 liquid handler; WaferGen; and library preparation kit, catalog number KK8201; Kapa Biosystems). Sheared DNA was end repaired to create 5′-phosphorylated, blunt-ended double-stranded DNA (dsDNA), and then adapters with 3′-deoxyribosylthymine (3′-dT) overhangs were ligated to the A-tailed library fragments (Bioo Scientific, catalog number 520999). Adapter-ligated DNA fragments were prepared for amplification using Kapa HiFi enzyme with AMPure beads (Agencourt Bioscience/Beckman Coulter, catalog number A63883). Libraries were checked for quality on an Agilent bioanalyzer and with quantitative PCR (library quantification kit, catalog number KK4835; Kapa). Samples were sequenced as 2 × 300-bp and 2 × 150-bp paired-end reads on an Illumina MiSeq platform. Read file data sets were combined, trimmed, partially assembled, and annotated using the Comprehensive Genome Analysis (CGA) feature of PATRIC version 3.5.26 (https://patricbrc.org), default parameters (2).

**FIG 1:**
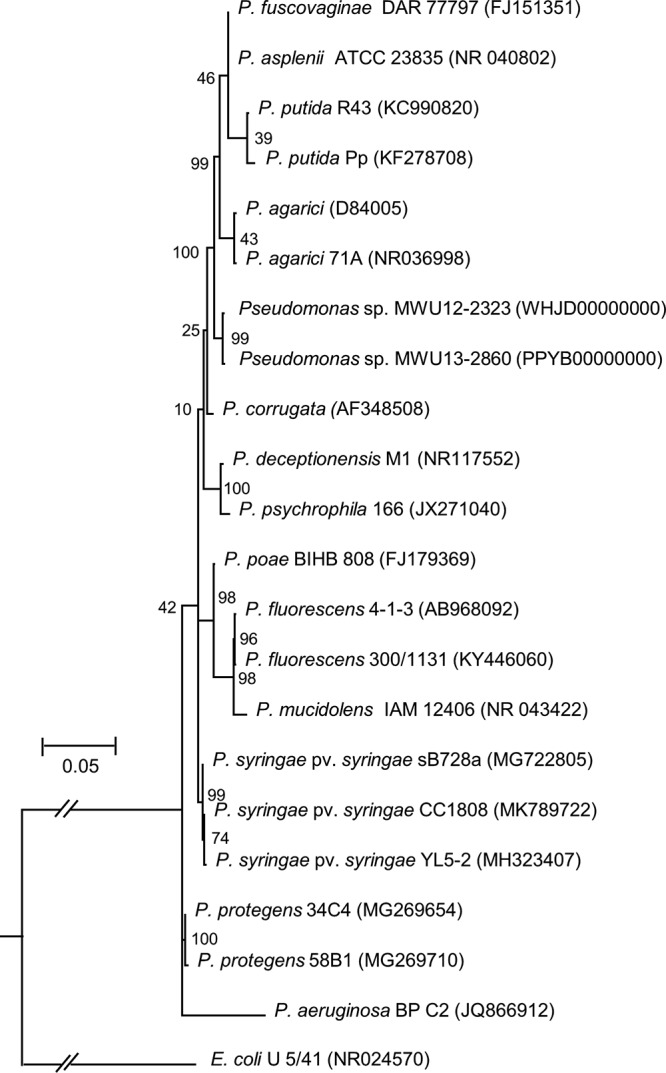
16S rRNA phylogeny of *Pseudomonas* sp. MWU12-2323. The evolutionary history of 22 selected members of the genus *Pseudomonas* was inferred using the maximum likelihood method based on the Kimura 2-parameter model (8), with discrete gamma distribution sites (5 categories [+G, parameter = 0.3066]). The rate variation model allowed some sites to be evolutionarily invariable ([+I], 64.1081% of sites), with complete deletion of gaps and missing data and a rate variation model that allowed for some evolutionarily invariable sites ([+I], 82.42% of sites). There was a total of 1,283 positions in the final data set. The tree with the highest log likelihood (–3,550.0097) is shown. Bootstrap values based on 500 samplings are shown at the nodes. Initial trees for the heuristic search were obtained by applying the neighbor-joining method to a matrix of pairwise distances estimated using the maximum composite likelihood (MCL) approach. Except for the Escherichia coli outgroup, the tree is drawn to scale, with branch lengths measured in the number of substitutions per site. Evolutionary analyses were conducted in MEGA6 (9). *Pseudomonas* sp. MWU12-2323 clusters with *Pseudomonas* sp. strain MWU13-2860, isolated from the same wild bog but taken in a different year. Accession numbers are shown in parentheses next to each organism name.

*Pseudomonas* sp. MWU12-2323 has an assembled genome size of 7,622,148 bp distributed over 67 contigs, with a 60.93% G+C content. Sequence coverage was 69.39× with an *N*_50_ value of 274,347 bp. MWU12-2323 clusters by 16S rRNA phylogeny with *Pseudomonas* sp. MWU13-2860 (3) but not with any named species (Fig. 1). High OrthoANI (4, 5) (98.16%) and digital DNA-DNA hybridization (dDDH) (6) (86.05%) values indicate that MWU12-2323 and MWU13-2860 are conspecific but not identical.

Functional annotation was performed using the Rapid Annotations using Subsystems Technologies tool kit (RAST*tk*) in the PATRIC CGA pipeline (7). There were 7,078 protein-coding genes and 62 tRNA and 5 rRNA operons, as well as genes encoding enzymes for the biosynthesis of nonribosomal protein sequences (NRPS), polyketides, pyoverdine, and bacteriocin, each of which has potential biocontrol activity against root pathogens. Genes related to polysaccharide-degrading enzymes, including those for cellulase, laccases, amylase, and chitinases, were also detected. The presence of genes for carbohydrate polymer-degrading enzymes and biologically active secondary metabolites may indicate a role as a natural suppressor of plant-pathogenic fungi.

### Data availability.

This whole-genome shotgun project has been deposited at DDBJ/EMBL/GenBank under the accession number WHJD00000000 for *Pseudomonas* sp. MWU12-2323. The Sequence Read Archive accession number is SRR10239425.

## References

[1] Gross H, Loper JE 2009 Genomics of secondary metabolite production by *Pseudomonas* spp. Nat Prod Rep 26:1408–1446. doi:10.1039/b817075b. 19844639

[2] Wattam AR, Davis JJ, Assaf R, Boisvert S, Brettin T, Bun C, Conrad N, Dietrich EM, Disz T, Gabbard JL, Gerdes S, Henry CS, Kenyon RW, Machi D, Mao C, Nordberg EK, Olsen GJ, Murphy-Olson DE, Olson R, Overbeek R, Parrello B, Pusch GD, Shukla M, Vonstein V, Warren A, Xia F, Yoo H, Stevens RL 2017 Improvements to PATRIC, the all-bacterial Bioinformatics Database and Analysis Resource Center. Nucleic Acids Res 45:D535–D542. doi:10.1093/nar/gkw1017. 27899627 PMC5210524

[3] Ebadzadsahrai G, Thomson J, Soby S 2018 Draft genome sequence of *Pseudomonas* sp. strain MWU13-2860, isolated from a wild cranberry bog in Truro, Massachusetts. Microbiol Resour Announc 7:e01007-18. doi:10.1128/MRA.01007-18. 30533681 PMC6256550

[4] Konstantinidis KT, Tiedje JM 2005 Genomic insights that advance the species definition for prokaryotes. Proc Natl Acad Sci U S A 102:2567–2572. doi:10.1073/pnas.0409727102. 15701695 PMC549018

[5] Yoon S-H, Ha S-m, Lim J, Kwon S, Chun J 2017 A large-scale evaluation of algorithms to calculate average nucleotide identity. Antonie Van Leeuwenhoek 110:1281–1286. doi:10.1007/s10482-017-0844-4. 28204908

[6] Meier-Kolthoff JP, Auch AF, Klenk H-P, Göker M 2013 Genome sequence-based species delimitation with confidence intervals and improved distance functions. BMC Bioinformatics 14:60. doi:10.1186/1471-2105-14-60. 23432962 PMC3665452

[7] Brettin T, Davis JJ, Disz T, Edwards RA, Gerdes S, Olsen GJ, Olson R, Overbeek R, Parrello B, Pusch GD, Shukla M, Thomason JA, Stevens R, Vonstein V, Wattam AR, Xia F 2015 RAST*tk*: a modular and extensible implementation of the RAST algorithm for building custom annotation pipelines and annotating batches of genomes. Sci Rep 5:8365. doi:10.1038/srep08365. 25666585 PMC4322359

[8] Kimura M 1980 A simple method for estimating evolutionary rates of base substitutions through comparative studies of nucleotide sequences. J Mol Evol 16:111–120. doi:10.1007/bf01731581. 7463489

[9] Tamura K, Stecher G, Peterson D, Filipski A, Kumar S 2013 MEGA6: Molecular Evolutionary Genetics Analysis version 6.0. Mol Biol Evol 30:2725–2729. doi:10.1093/molbev/mst197. 24132122 PMC3840312

